# Supplementary material for: Synthesis and Properties of Pentafluorosulfanyl Group (SF5)-Containing Meta-Diamide Insecticides
Source: Molecules. 2020 Nov 25;25(23):5536. doi: 10.3390/molecules25235536 (PMC7728329; doi:10.3390/molecules25235536)
Supplement: Supplementary file 1 [file molecules-25-05536-s001.pdf]

molecules

MDPI

Communication

Synthesis and Properties of Pentafluorosulfanyl Group (SF_5)-Containing meta-Diamide Insecticides

Jae Gon Kim [1], On-Yu Kang [1,2], Sang Mee Kim [1], Guldana Issabayeva [1,3], In Seok Oh [1,4], Yaeji Lee [1,3], Won Hyung Lee [5], Hwan Jung Lim [1,3,*] and Seong Jun Park [1,*]

1 Bio & Drug Discovery Division, Korea Research Institute of Chemical Technology (KRICT), 141 Gajeong-ro, Yuseong-gu, Daejeon 34114, Korea; jgkim@krict.re.kr (J.G.K.); dhdb0901@krict.re.kr (O.-Y.K.); smk95@krict.re.kr (S.M.K.); guldana@krict.re.kr (G.I.); ois0821@krict.re.kr (I.S.O.); leeyj@krict.re.kr (Y.L.)

2 Department of Chemistry, Sungkyunkwan University, Suwon 16419, Korea

3 Department of Medicinal and Pharmaceutical Chemistry, University of Science & Technology, Daejeon 34113, Korea

4 Department of Chemistry, Sogang University, Seoul 04107, Korea

5 Central Research Institute, Kyung Nong Co. Ltd., 34-14 Summeori-gil, Kyongju 38175, Korea; whlee1@dongoh.co.kr

* Correspondence: indium@krict.re.kr (H.J.L.); sjunpark@krict.re.kr (S.J.P.); Tel.: +82-42-860-7177 (H.J.L.); +82-42-860-7175 (S.J.P.)

Academic Editors: György Szöllösi and Eva Frank

Received: 21 October 2020; Accepted: 23 November 2020; Published: 25 November 2020

Supporting information (Supplementary Data)

Contents

Result & data

^{1}H, ^{13}C and ^{19}F NMR spectroscopy of **1c**. 2

^{1}H, ^{13}C and ^{19}F NMR spectroscopy of **1d**. 4

^{1}H, ^{13}C and ^{19}F NMR spectroscopy of **3**. 6

^{1}H, ^{13}C and ^{19}F NMR spectroscopy of **4a**. 8

^{1}H, ^{13}C and ^{19}F NMR spectroscopy of **4b**. 10

^{1}H, ^{13}C and ^{19}F NMR spectroscopy of **4c**. 12

^{1}H, ^{13}C and ^{19}F NMR spectroscopy of **4d**. 14

Table S1 and S2: Larvicidal activity against Plutella xylostella (4c and 4d) 16

Figure S1: pH-metric Log P of compounds **4d** (KI-03066) 20

Figure S2: pH-metric Log P of **Broflanilide** 23

Figure S3. Ion Channels assay of **4d** (KI-03066) and Broflanilide 24

^{1}H, ^{13}C and ^{19}F NMR spectroscopy of 1c.

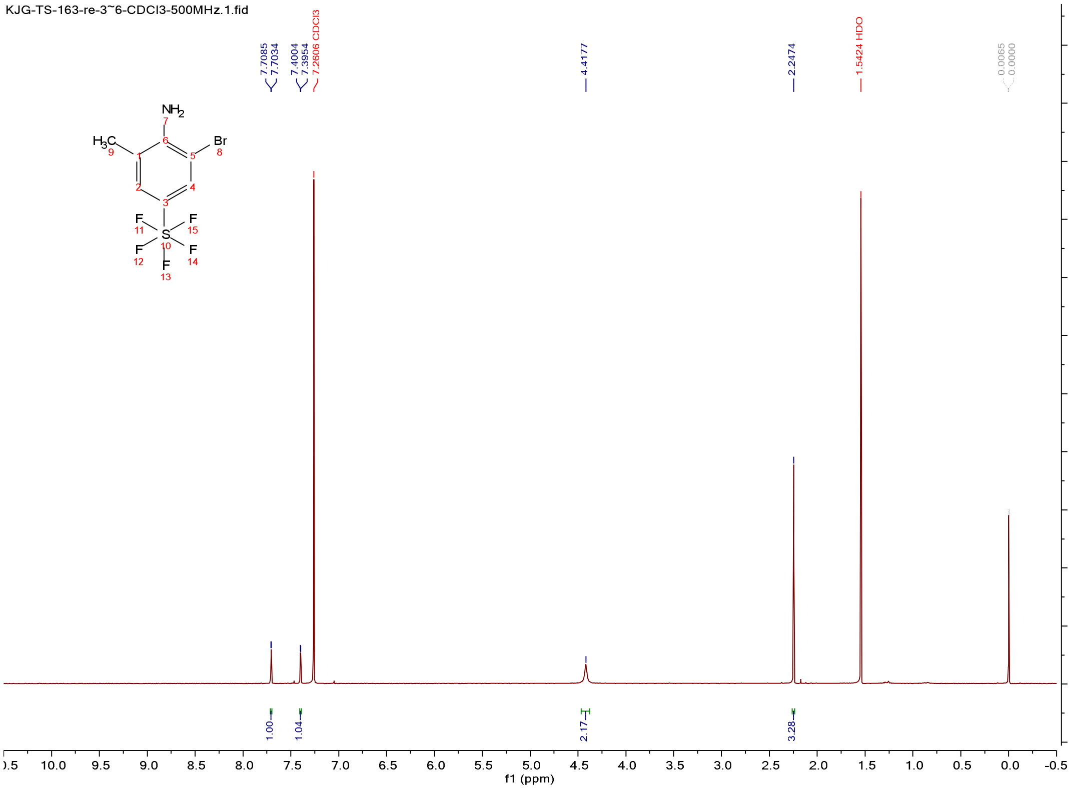

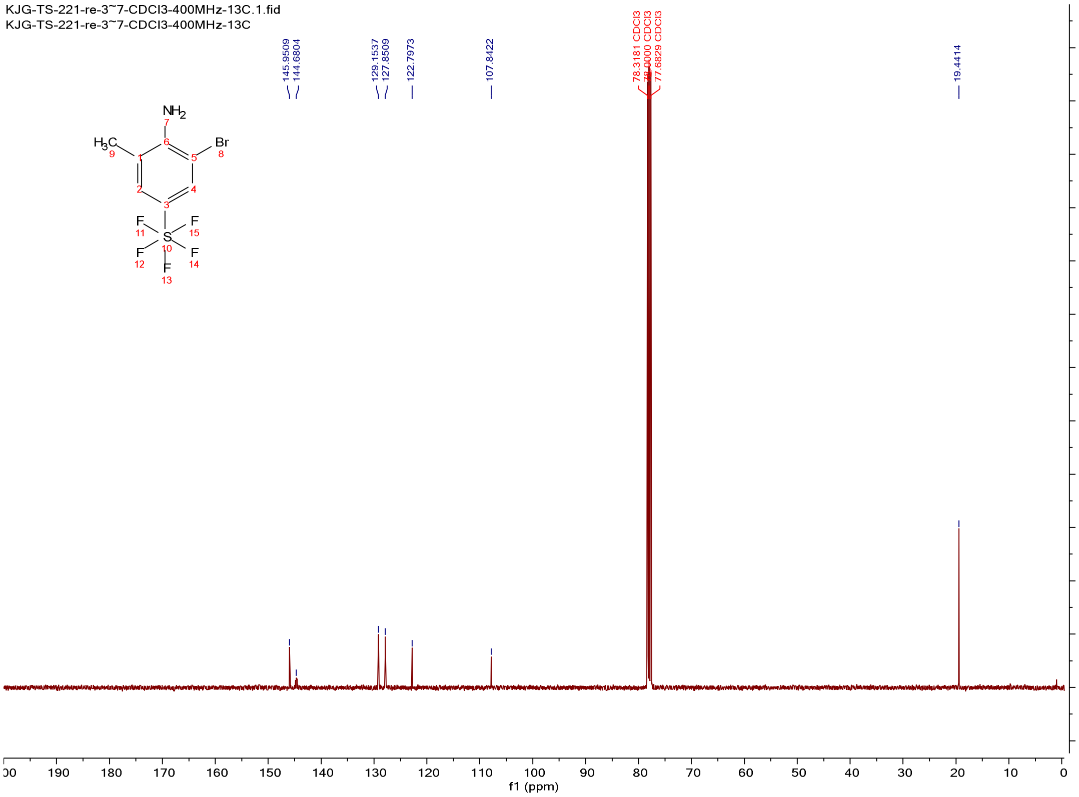

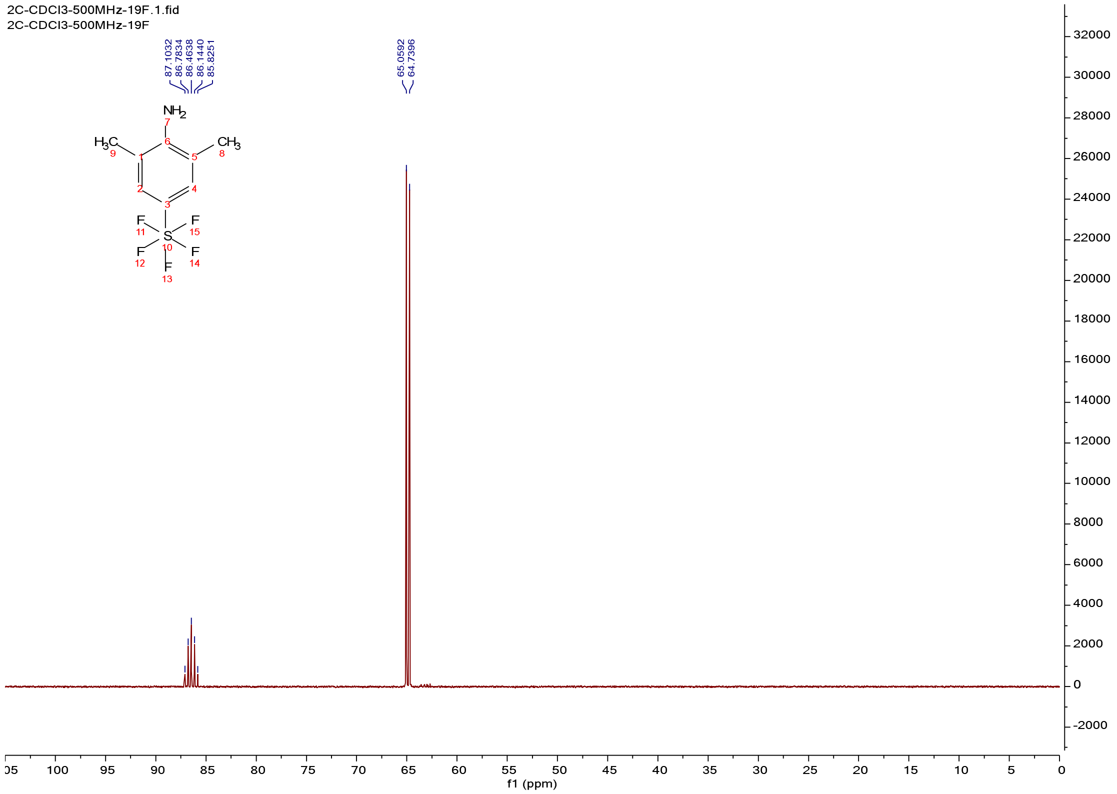

2C-CDCl3-500MHz-19F.1.fid
2C-CDCl3-500MHz-19F
87.1032
86.7834
86.4638
86.1440
85.8251
65.0592
64.7396
f1 (ppm)

^1H, ^{13}C and ^{19}F NMR spectroscopy of 1d.

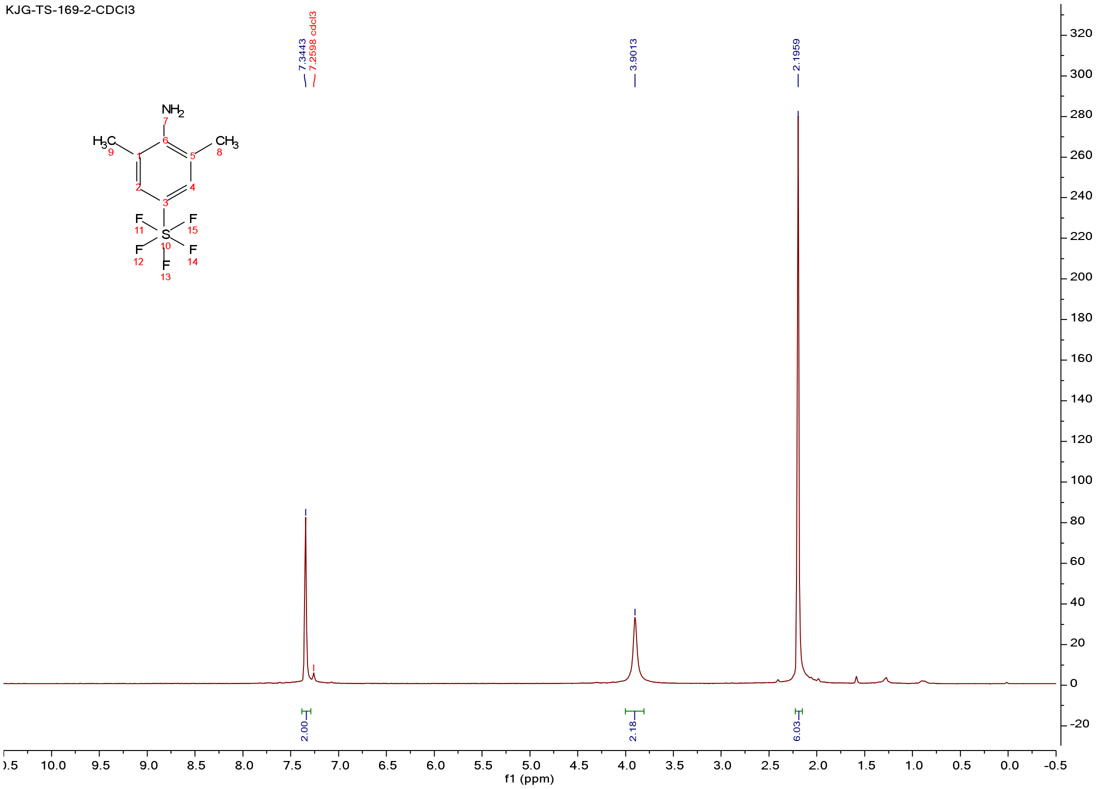

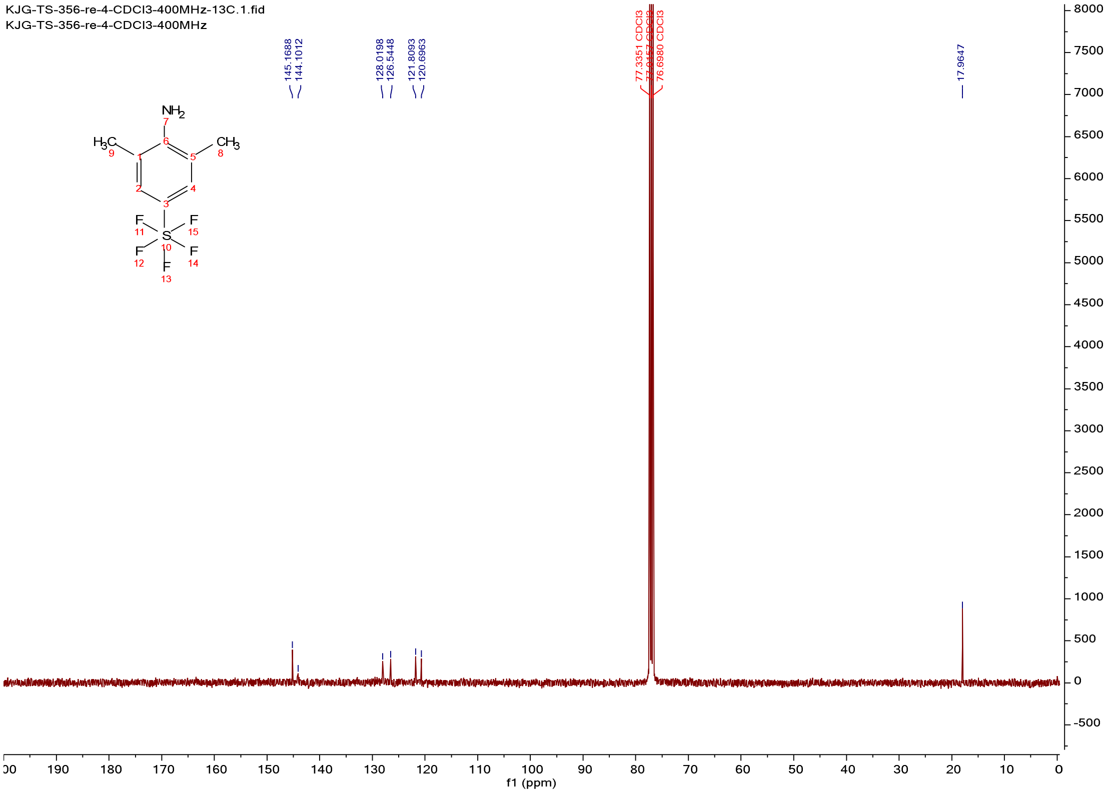

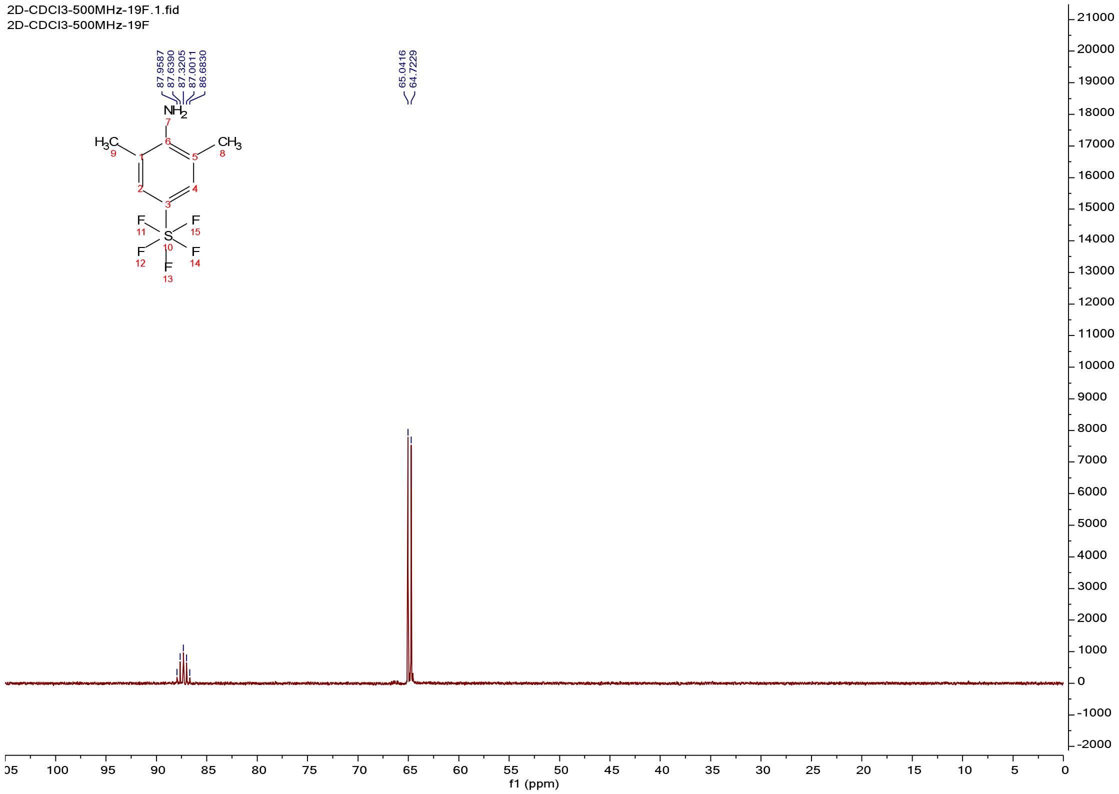
2D-CDCl3-500MHz-19F.1.fid
2D-CDCl3-500MHz-19F
87.9587
87.6390
87.3205
87.0011
86.6830
65.0416
64.7229
f1 (ppm)

^{1}H, ^{13}C and ^{19}F NMR spectroscopy of 3.

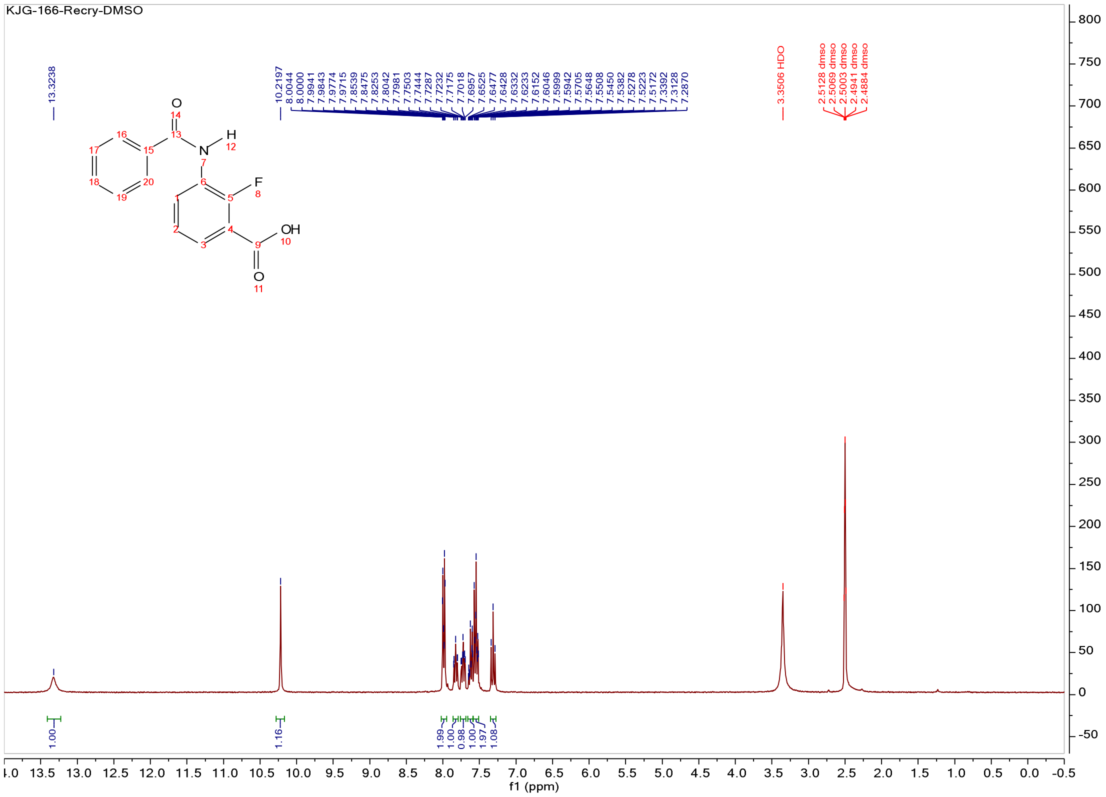

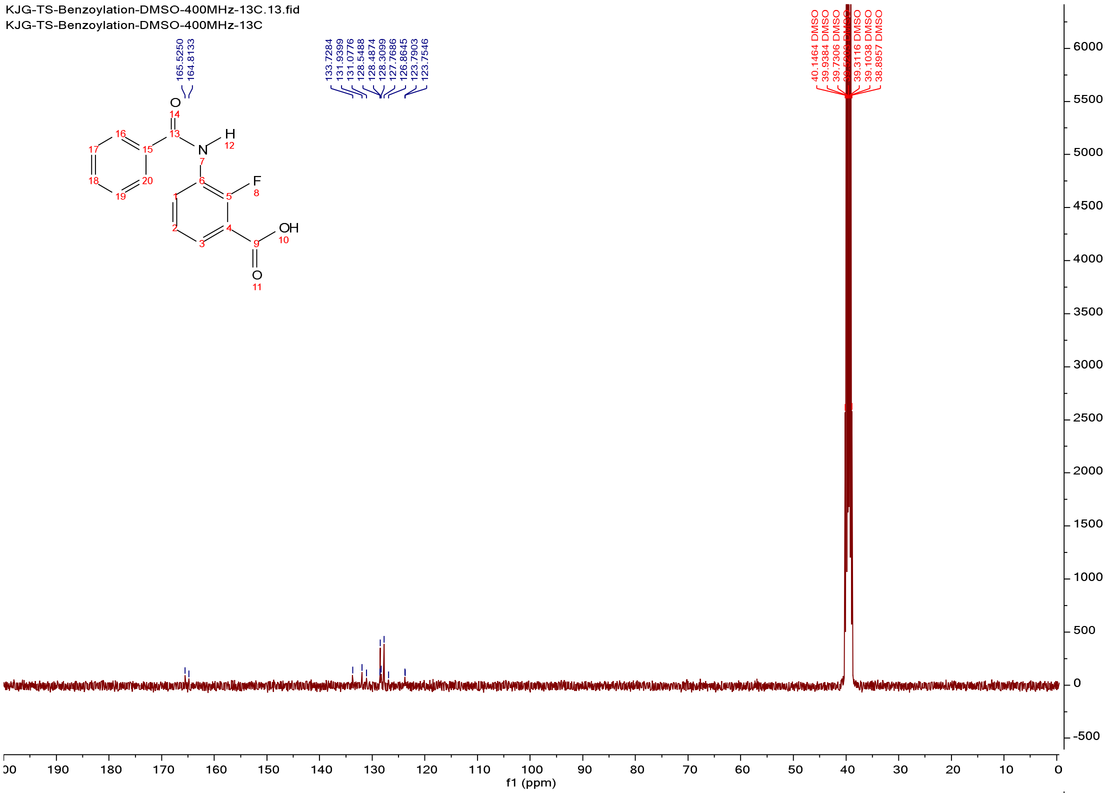
KJG-TS-Benzoylation-DMSO-400MHz-13C.13.fid
KJG-TS-Benzoylation-DMSO-400MHz-13C
f1 (ppm)

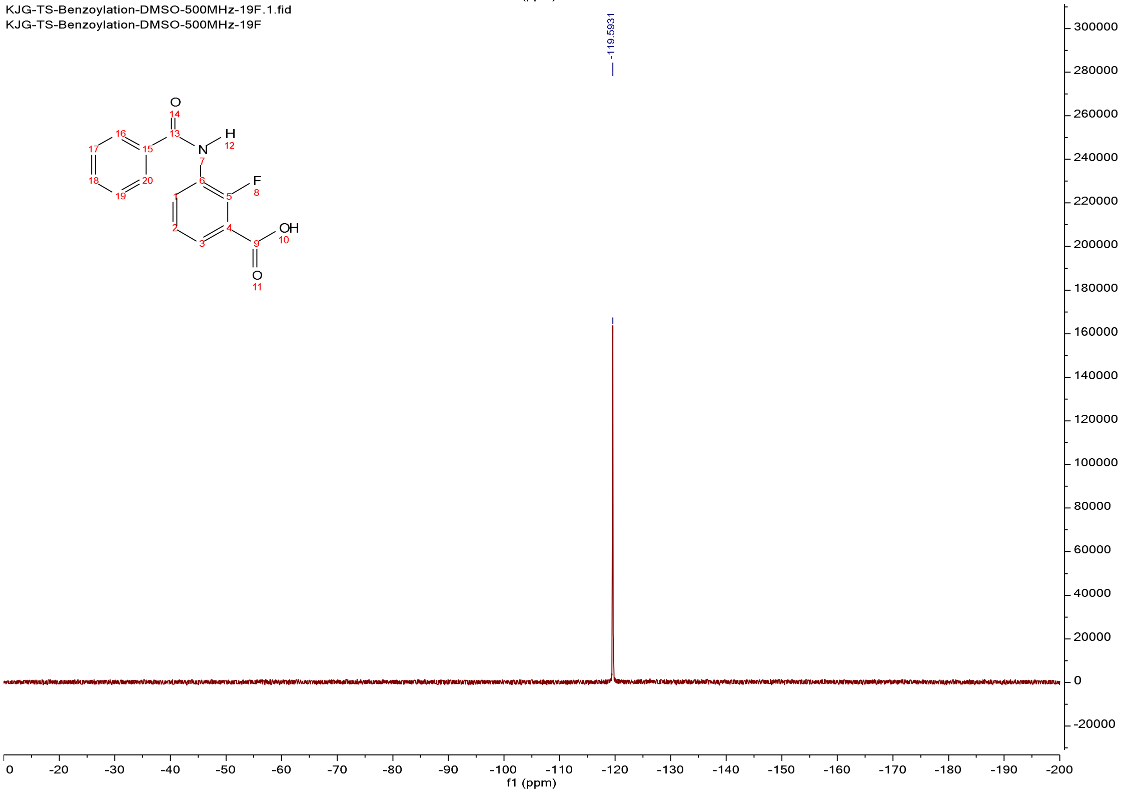
KJG-TS-Benzoylation-DMSO-500MHz-19F.1.fid
KJG-TS-Benzoylation-DMSO-500MHz-19F
-119.5931
f1 (ppm)

^{1}H, ^{13}C and ^{19}F NMR spectroscopy of 4a.

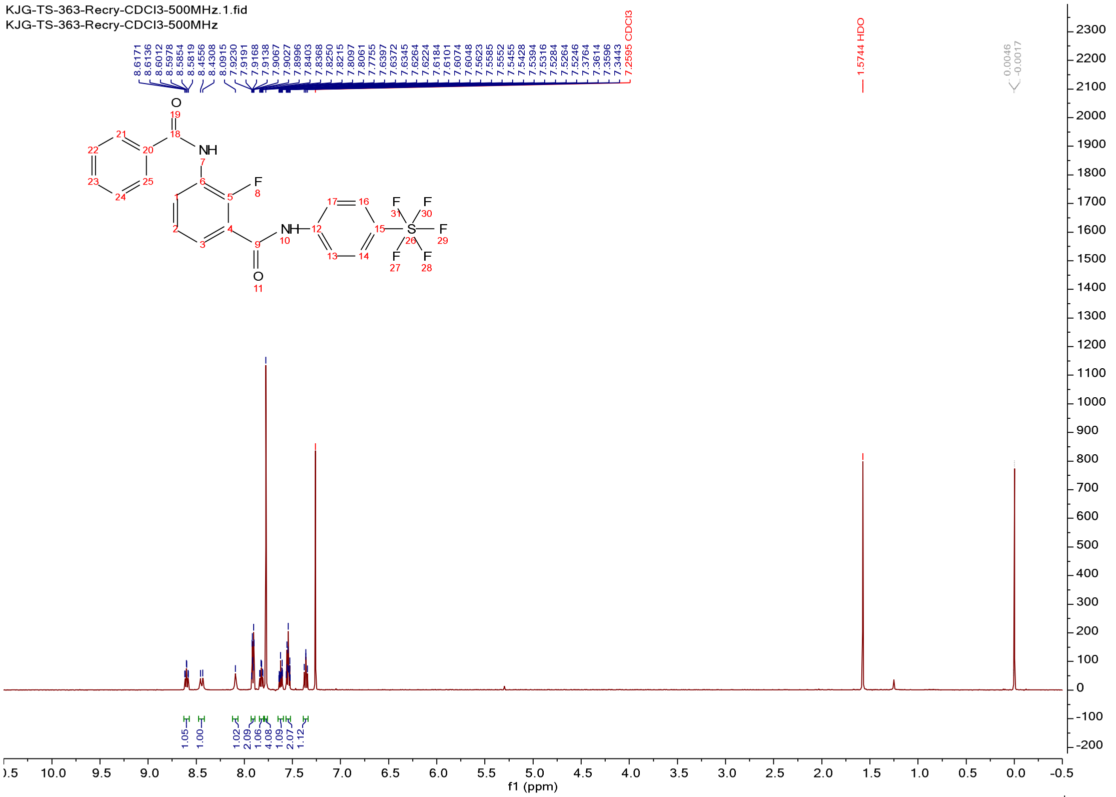

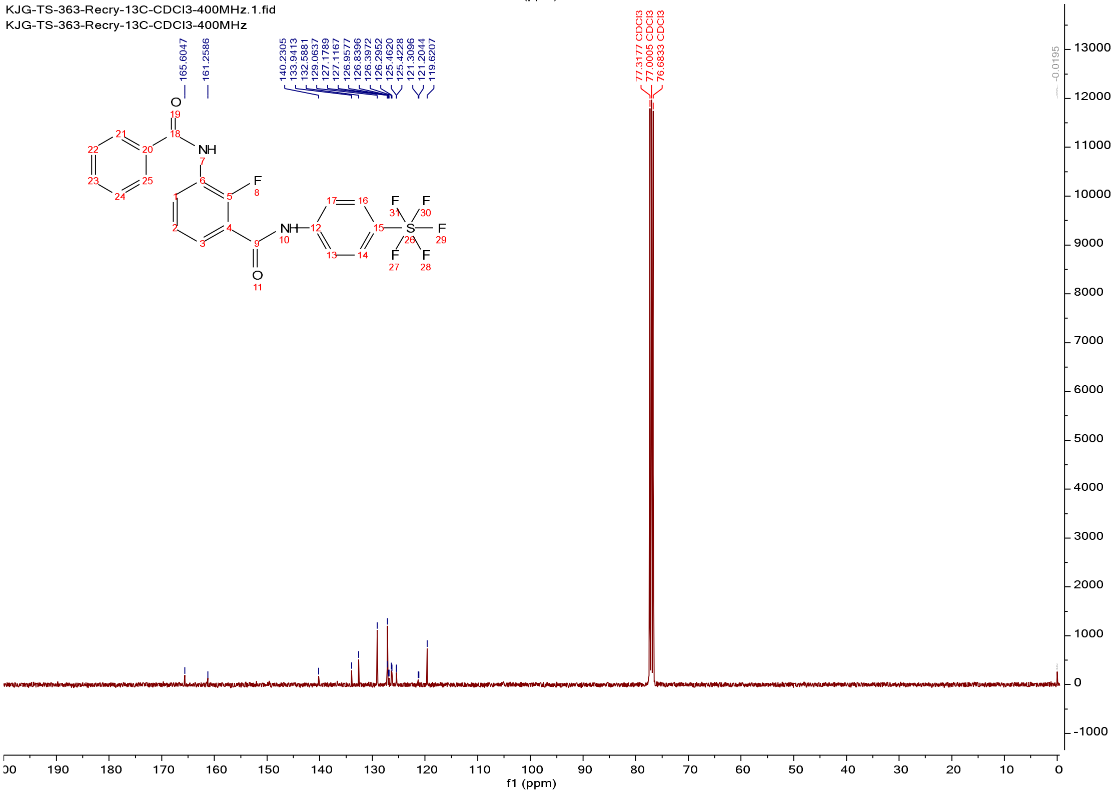

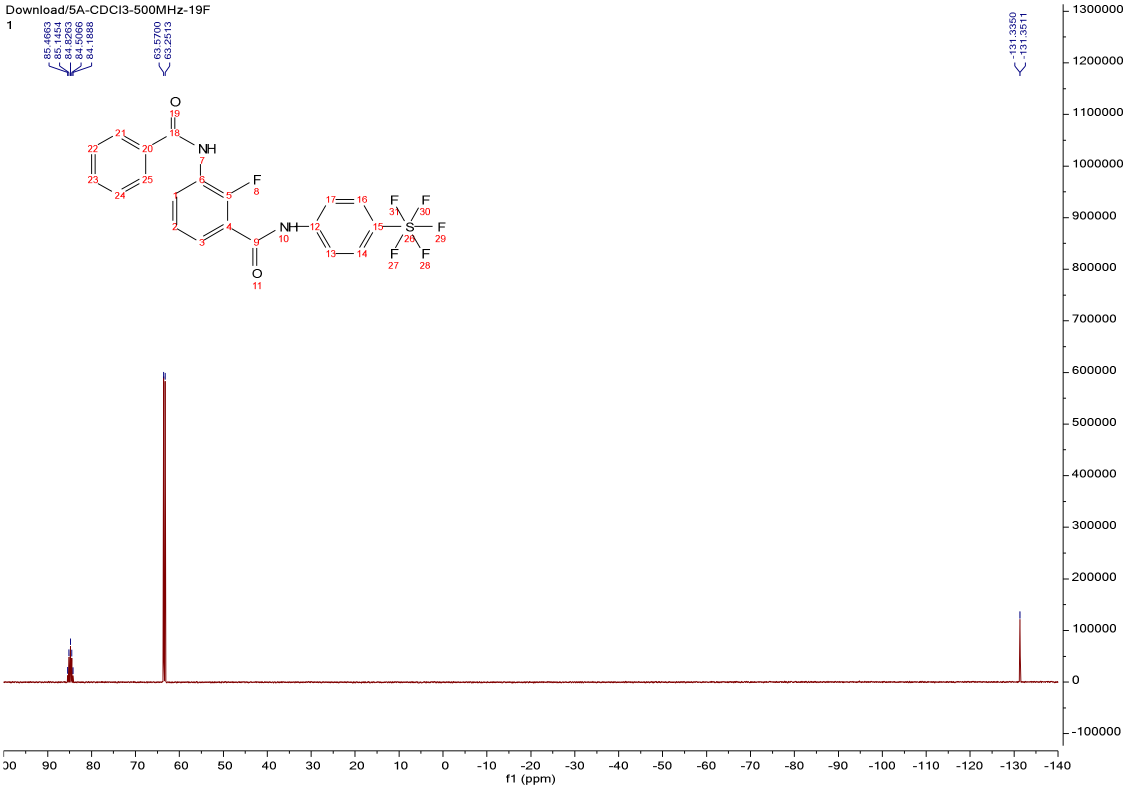
Download/5A-CDCl3-500MHz-19F
1
85.4663
85.1454
84.8263
84.5066
84.1888
63.5700
63.2513
-131.3350
-131.3511
f1 (ppm)

^{1}H, ^{13}C and ^{19}F NMR spectroscopy of 4b.

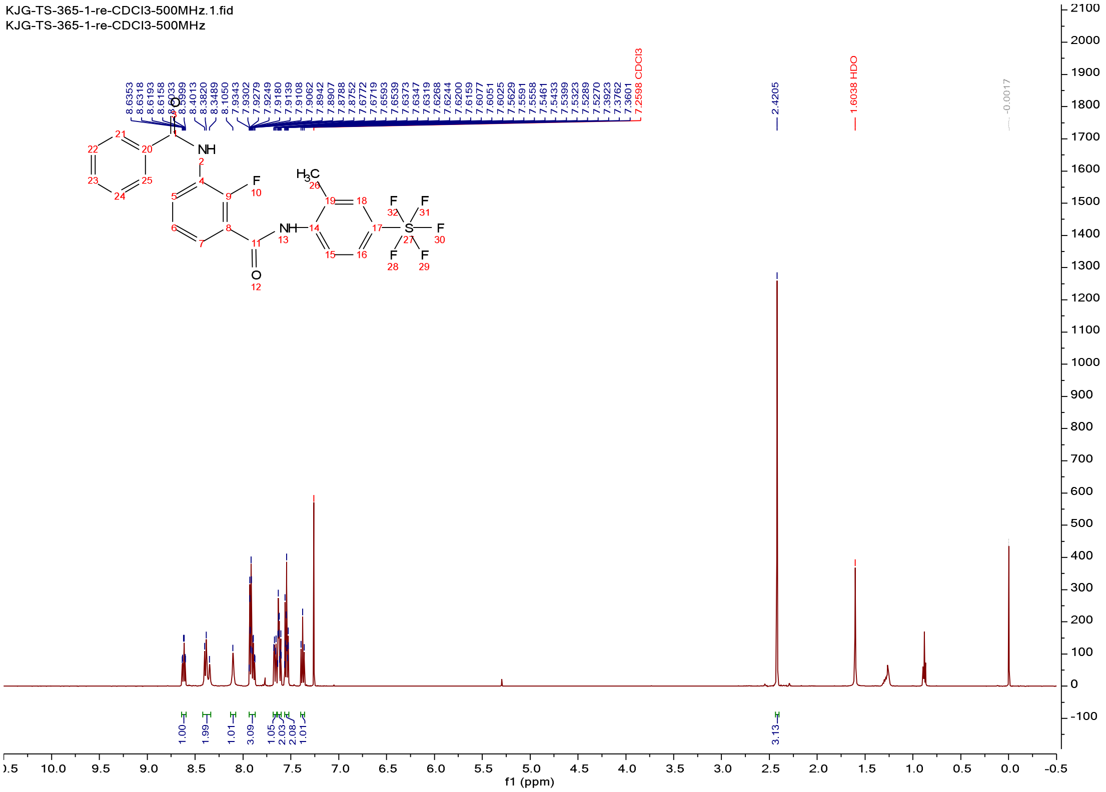

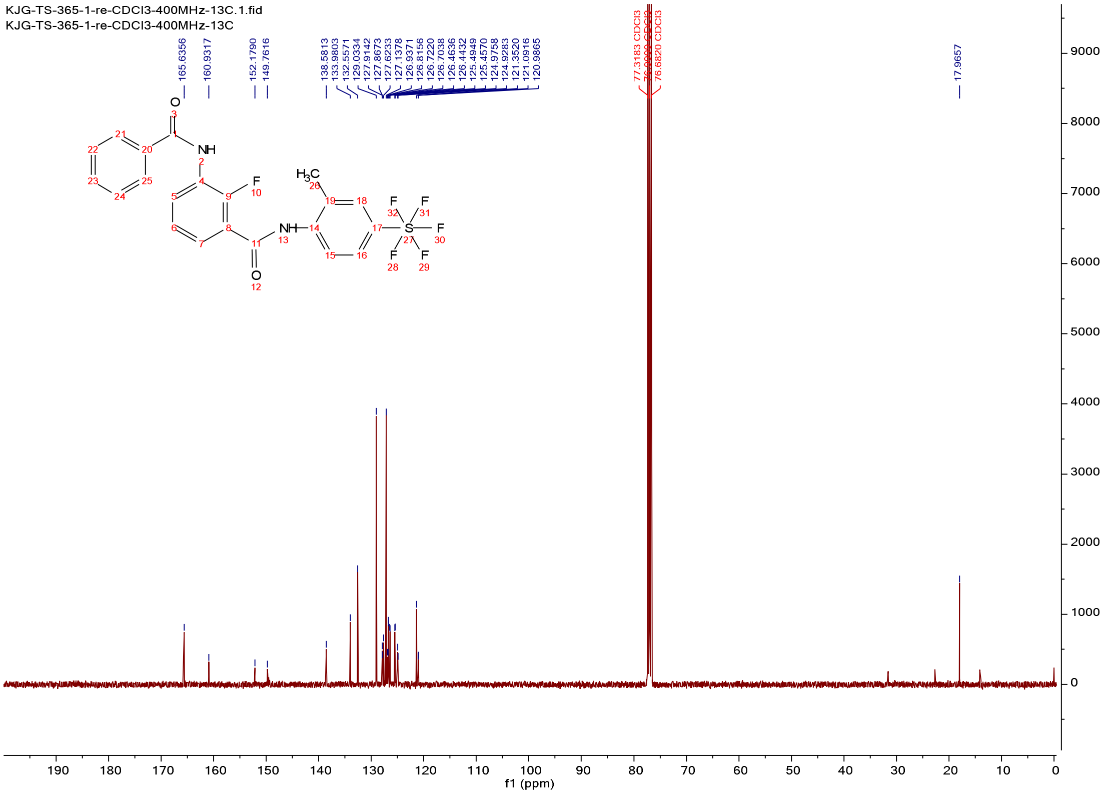

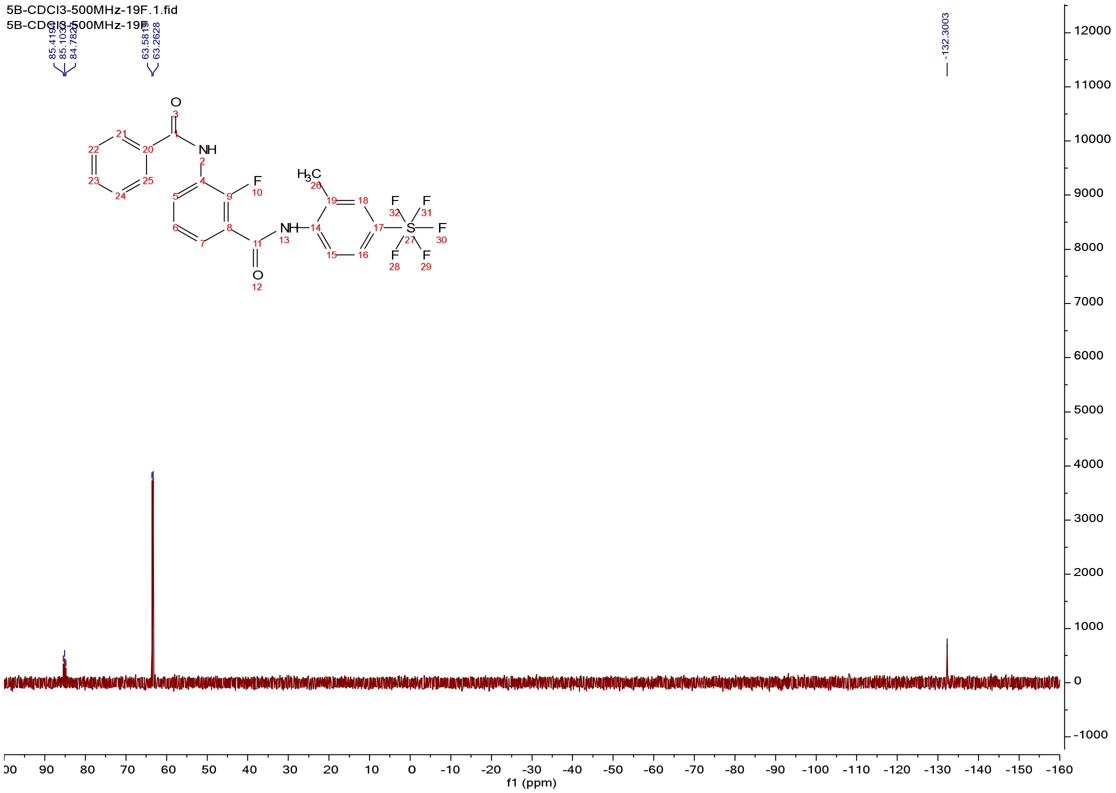
5B-CDCl3-500MHz-19F.1.fid
5B-CDCl3-500MHz-19F
85.4193
85.1032
84.7824
63.5819
63.2628
-132.3003
f1 (ppm)

^{1}H, ^{13}C and ^{19}F NMR spectroscopy of 4c.

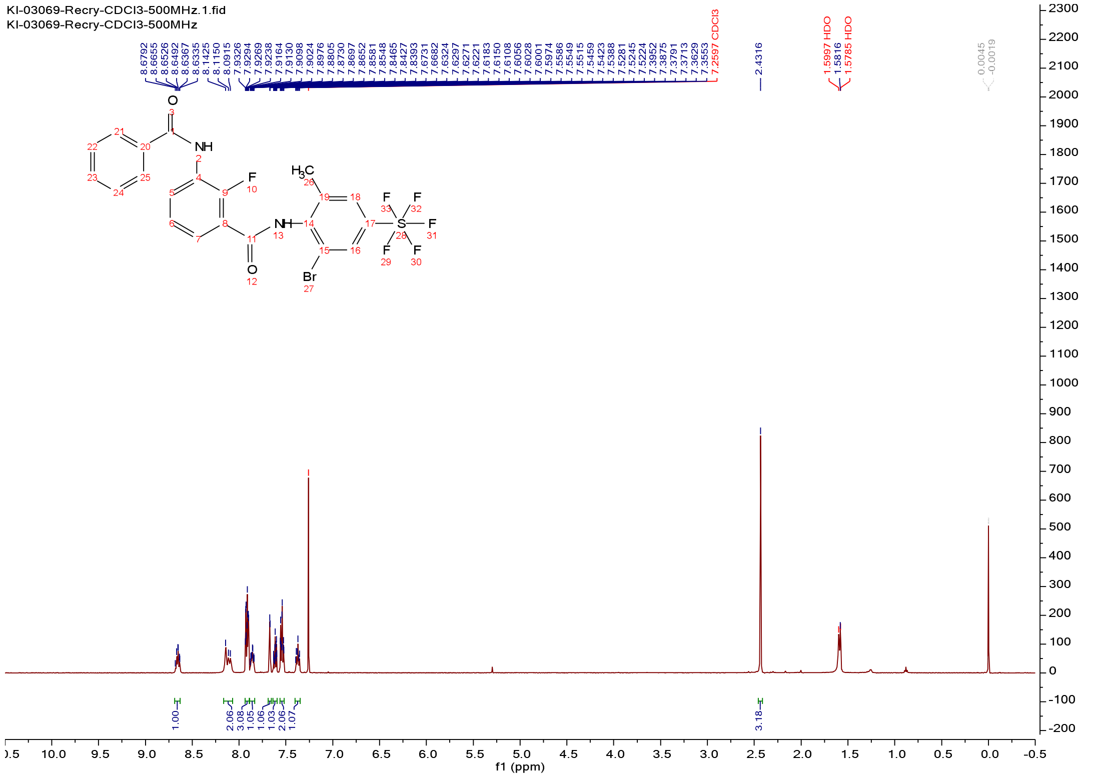

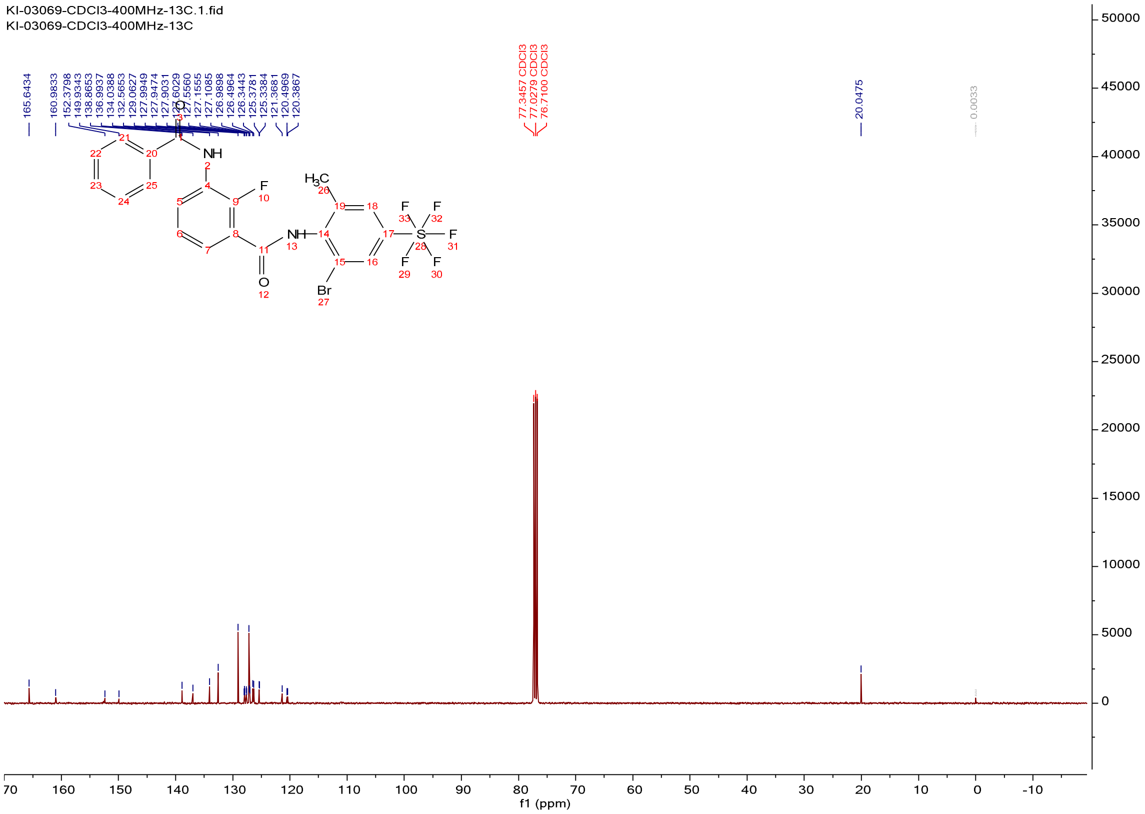

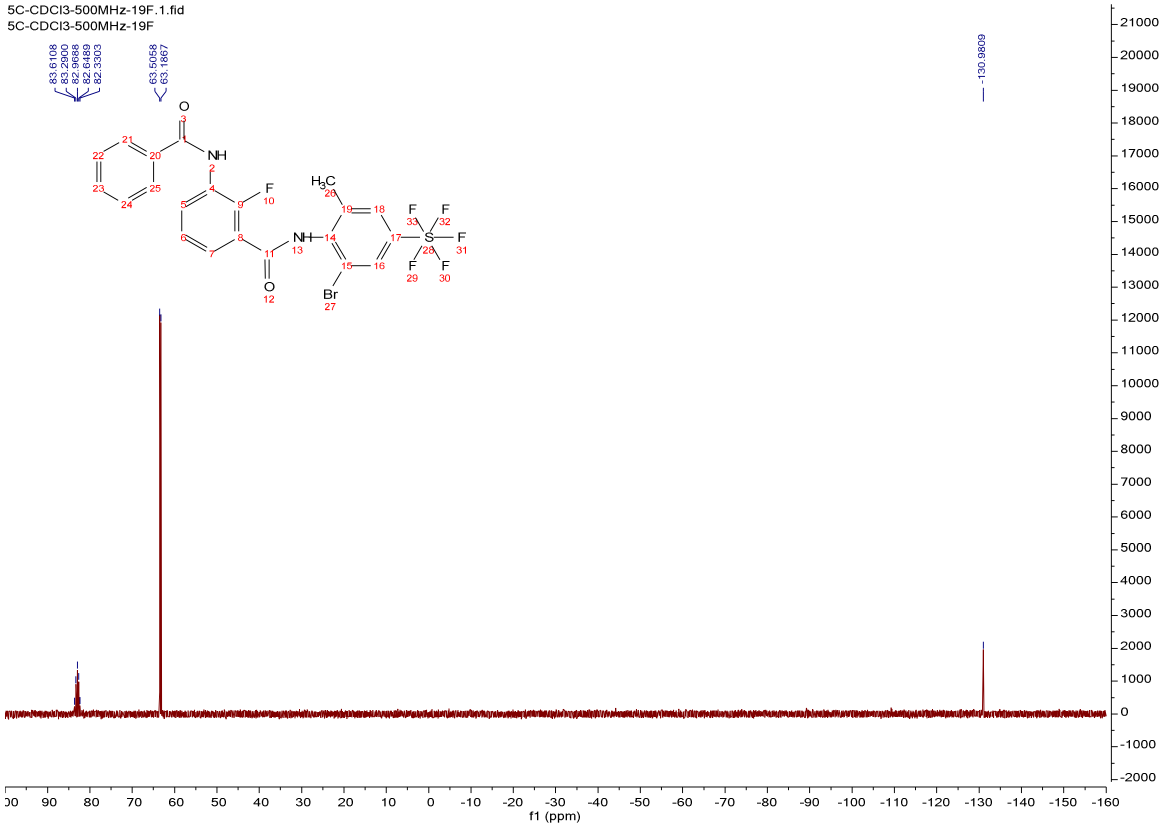

5C-CDCl3-500MHz-19F.1.fid
5C-CDCl3-500MHz-19F
83.6108
83.2900
82.9688
82.6489
82.3303
63.5058
63.1867
-130.9809
f1 (ppm)

^{1}H, ^{13}C and ^{19}F NMR spectroscopy of 4d.

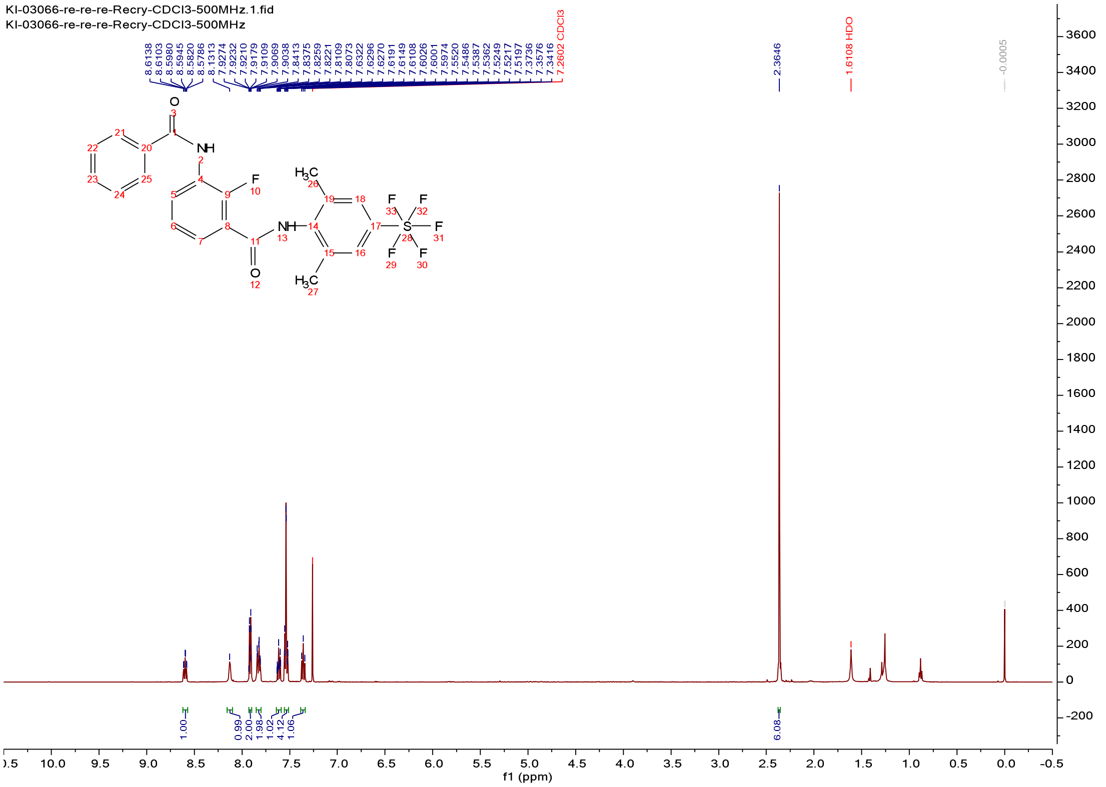

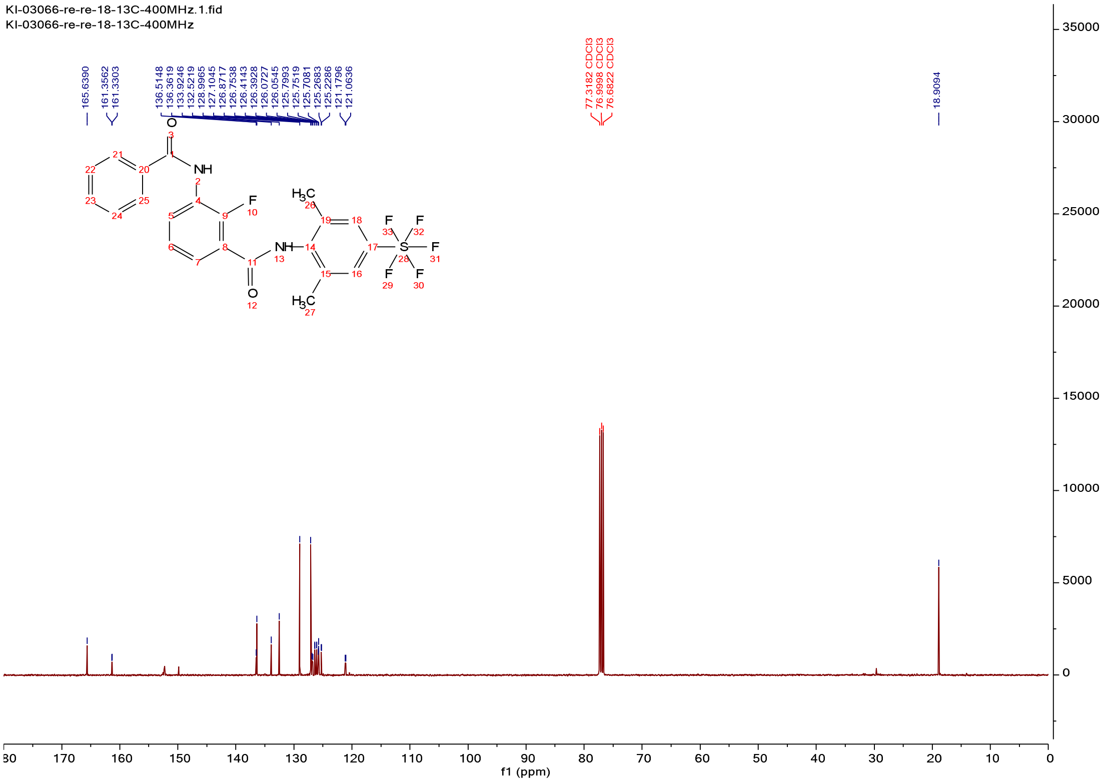

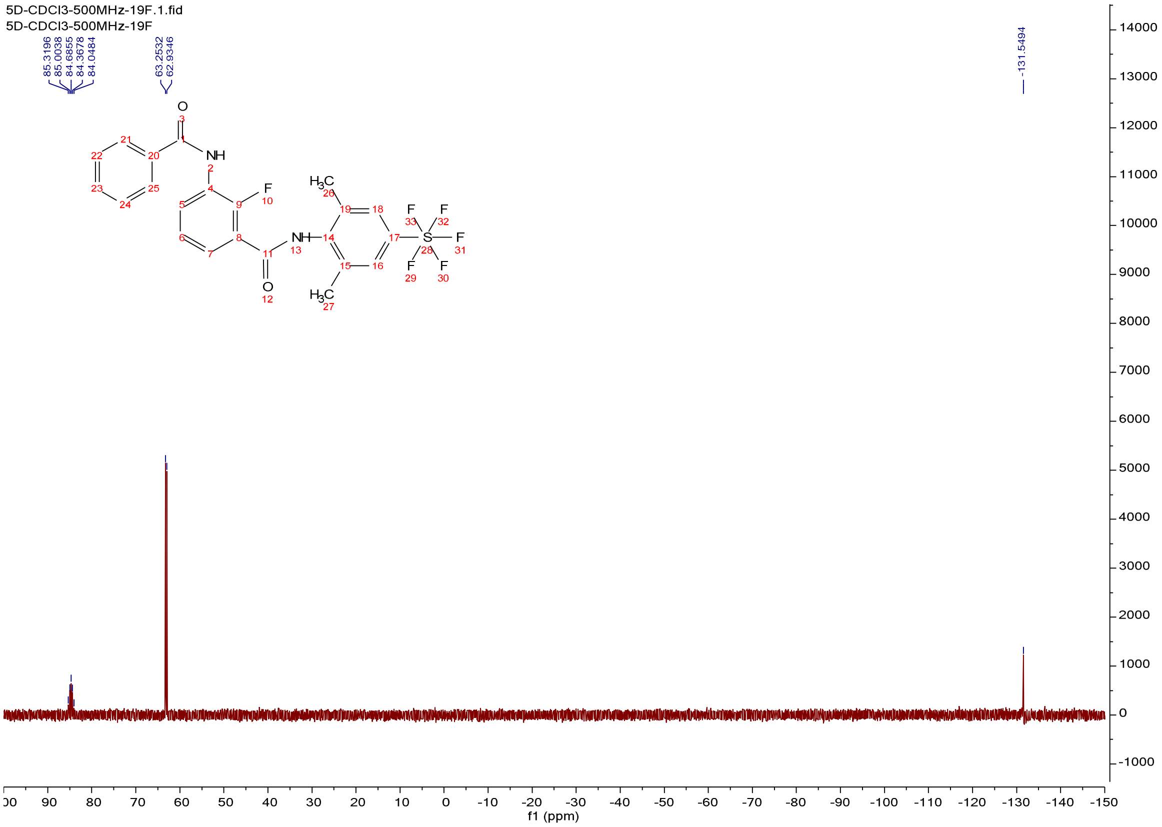
5D-CDCl3-500MHz-19F.1.fid
5D-CDCl3-500MHz-19F
85.3196
85.0038
84.6855
84.3678
84.0484
63.2532
62.9346
-131.5494
f1 (ppm)

Table S1 and S2: Larvicidal activity against Plutella xylostella (4c and 4d)

The lavicidal activities of synthesized compounds were evaluated by the leaf-dip procedure. The aqueous solution of prepared compounds and Broflanilide in acetone (H_2O : acetone = 95 : 5) were sprayed to a cabbage leave placed on moistened filter paper (disc, diameter 8.8 cm) in petri dishes. After allowing to dry, the dishes were infested with 10 Plutella xylostella (third-instar). After 1, 2, 3, and 4 days, percentage of mortalities was evaluated. The treatments were replicated three times. For negative control, larvicidal activities were 0 % at each time. Ref. is Broflanilide as a positive control.

Table S1. Larvicidal activity depend on time.

Entry	Compound	Concentration (ppm)	Against the 3rd Instar Larvae of *Plutella xylostella*			
			Larvicidal activity (%) at **24 h**			
			1	2	3	Average
1	**4c**	10	50.0	60.0	60.0	56.7
2	**4d**	10	0	10.0	20.0	10.0
3	Ref.	10	100	100	100	100.0

Entry	Compound	Concentration (ppm)	Against the 3rd Instar Larvae of *Plutella xylostella*			
			Larvicidal activity (%) at 48 h			
			1	2	3	Average
1	**4c**	10	90.0	90.0	90.0	90.0
2	**4d**	10	90.0	70.0	80.0	80.0
4	Ref.	10	100	100	100	100.0

Entry	Compound	Concentration (ppm)	Against the 3rd Instar Larvae of *Plutella xylostella*			
			Larvicidal activity (%) at 72 h			
			1	2	3	Average
1	**4c**	10	90.0	90.0	90.0	90.0
2	**4d**	10	90.0	80.0	80.0	83.3
4	Ref.	10	100	100	100	100.0

Entry	Compound	Concentration (ppm)	Against the 3rd Instar Larvae of *Plutella xylostella*			
			Larvicidal activity (%) at 96 h			
			1	2	3	Average
1	**4c**	10	90.0	90.0	90.0	90.0
2	**4d**	10	90.0	90.0	80.0	86.7
4	Ref.	10	100	100	100	100.0

Table S2. Picture of eating area.

Entry	Compd	Pictures of eating area_ after 96 h (The 3rd instar stage larvae of *Spodoptera litura*)
1	**4c**	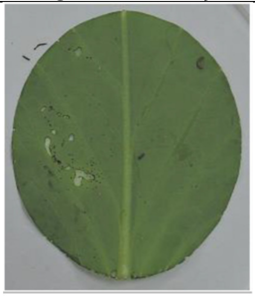 5–10 % eating
2	**4d**	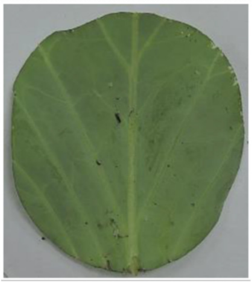 0–5 % eating
3	Ref. positive control (Broflanilide)	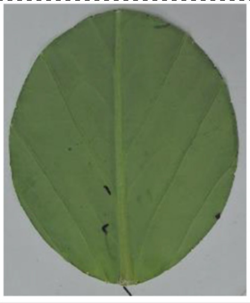 0–5 % eating
4	Negative control	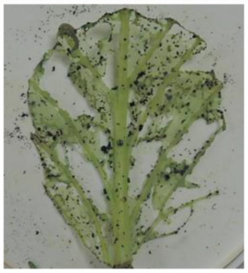

pH-metric

Sample name:	**KI-03066**	Experiment start time:	**08/09/2020 07:26:11**
Assay name:	**pH-metric medium logP**	Analyst:	
Assay ID:	**20I-08012**	Instrument ID:	**T313101**
Filename:	**D:\Data\Customer\20I-08012_KI-03066_pH-metric medium logP.t3r**		

Overall results

RMSD	0.168
Average ionic strength	0.156 M
Average temperature	25.0°C
Partition ratio	0.2833 : 1
Analyte concentration range	1430.9 μM to 1492.8 μM
Total points considered	30 of 44

Warnings and errors

Errors	None
Warnings	Sample concentration factor out of range

Four-Plus parameters

Alpha	0.072	08/09/2020 07:26:11	D:\Data\Customer\20I-07022_Blank standardisation.t3r
S	1.0038	08/09/2020 07:26:11	D:\Data\Customer\20I-07022_Blank standardisation.t3r
jH	0.4	08/09/2020 07:26:11	D:\Data\Customer\20I-07022_Blank standardisation.t3r
jOH	-0.3	08/09/2020 07:26:11	D:\Data\Customer\20I-07022_Blank standardisation.t3r

Titrants

0.50 M HCl	0.979835	08/09/2020 07:26:11	D:\Data\Customer\20I-07023_Blank standardisation.t3r
0.50 M KOH	1.004720	08/09/2020 07:26:11	D:\Data\Customer\20I-07020_KHP_Base standardisation using KHP.t3r

Sample

KI-03066 concentration factor	0.088
Acid pKa 1	11.00
Acid pKa 2	12.20
logP (neutral XH2)	4.68
logP (XH -)	6.11
logP (X 2-)	-3.45

Sample graphs

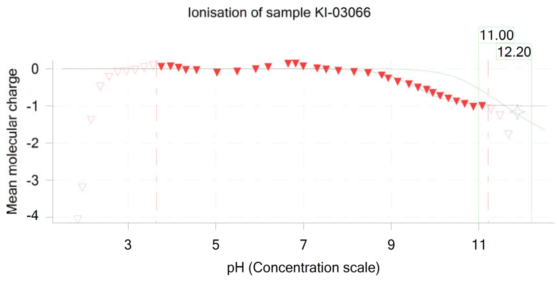

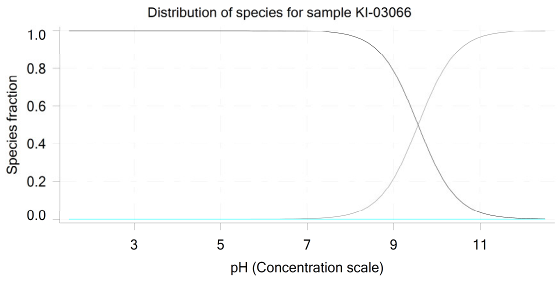

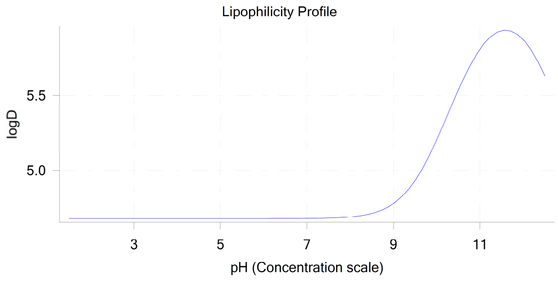

pH-metric

Sample name:	**KI-03066**	Experiment start time:	**08/09/2020 07:26:11**
Assay name:	**pH-metric medium logP**	Analyst:	
Assay ID:	**20I-08012**	Instrument ID:	**T313101**
Filename:	**D:\Data\Customer\20I-08012_KI-03066_pH-metric medium logP.t3r**		

Sample logD and percent species

pH	KI-03066 logD	KI-03066 KI-03066H2	KI-03066 KI-03066H	KI-03066 KI-03066	KI-03066 KI-03066H2*	KI-03066 KI-03066H*	KI-03066 KI-03066*	Comment
1.000	4.68	0.01 %	0.00 %	0.00 %	99.99 %	0.00 %	0.00 %	
1.200	4.68	0.01 %	0.00 %	0.00 %	99.99 %	0.00 %	0.00 %	Stomach pH
2.000	4.68	0.01 %	0.00 %	0.00 %	99.99 %	0.00 %	0.00 %	
3.000	4.68	0.01 %	0.00 %	0.00 %	99.99 %	0.00 %	0.00 %	
4.000	4.68	0.01 %	0.00 %	0.00 %	99.99 %	0.00 %	0.00 %	
5.000	4.68	0.01 %	0.00 %	0.00 %	99.99 %	0.00 %	0.00 %	
6.000	4.68	0.01 %	0.00 %	0.00 %	99.97 %	0.03 %	0.00 %	
6.500	4.68	0.01 %	0.00 %	0.00 %	99.91 %	0.09 %	0.00 %	
7.000	4.68	0.01 %	0.00 %	0.00 %	99.72 %	0.27 %	0.00 %	
7.400	4.68	0.01 %	0.00 %	0.00 %	99.31 %	0.68 %	0.00 %	Blood pH
8.000	4.69	0.01 %	0.00 %	0.00 %	97.35 %	2.65 %	0.00 %	
9.000	4.78	0.01 %	0.00 %	0.00 %	78.62 %	21.37 %	0.00 %	
10.000	5.20	0.00 %	0.00 %	0.00 %	26.89 %	73.10 %	0.00 %	
11.000	5.81	0.00 %	0.00 %	0.00 %	3.55 %	96.45 %	0.00 %	
12.000	5.87	0.00 %	0.00 %	0.00 %	0.37 %	99.63 %	0.00 %	

Carbonate and acidity

Carbonate	0.138 mM
Acidity error	0.539 mM

Other graphs

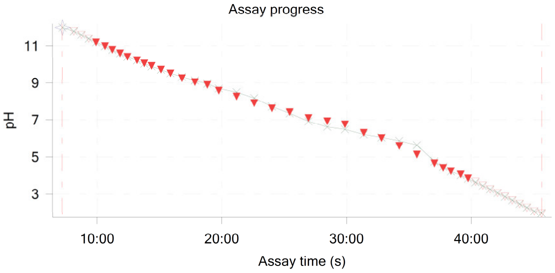

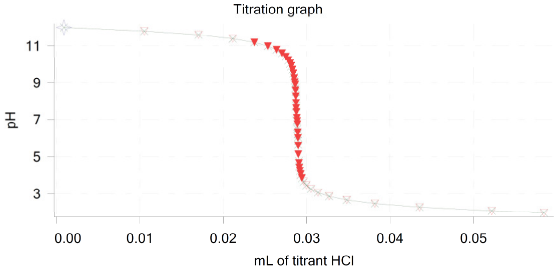

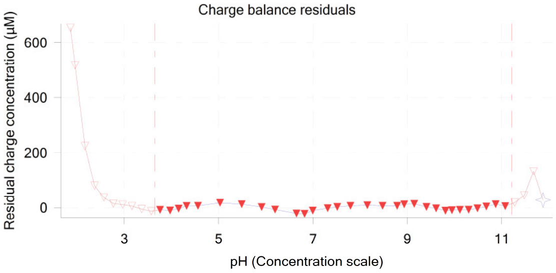

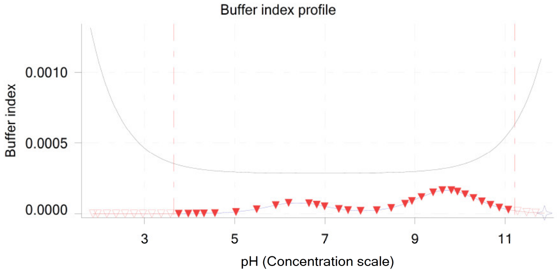

pH-metric

Sample name: **KI-03066**
Assay name: **pH-metric medium logP**
Assay ID: **20I-08012**
Filename: **D:\Data\Customer\20I-08012_KI-03066_pH-metric medium logP.t3r**

Experiment start time: **08/09/2020 07:26:11**
Analyst:
Instrument ID: **T313101**

Other graphs (continued)

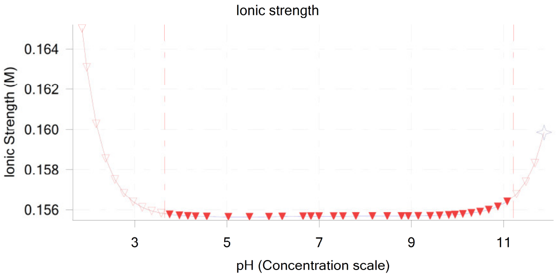

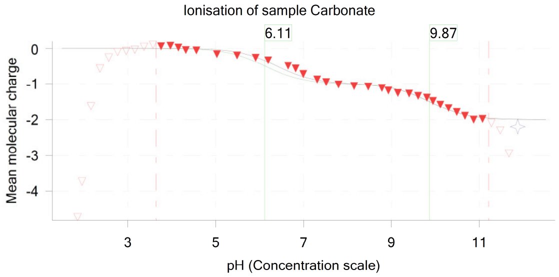

Figure S1. pH-metric Log P of compounds **4d** (KI-03066).

pH-metric

Sample name:	**KI-ref-B**	Experiment start time:	**08/09/2020 06:53:17**
Assay name:	**pH-metric medium logP**	Analyst:	
Assay ID:	**20I-08011**	Instrument ID:	**T313101**
Filename:	**D:\Data\Customer\20I-08011_KI-ref-B_pH-metric medium logP.t3r**		

Overall results

RMSD	0.157
Average ionic strength	0.156 M
Average temperature	25.0°C
Partition ratio	0.2834 : 1
Analyte concentration range	1063.3 µM to 1110.3 µM
Total points considered	21 of 32

Warnings and errors

Errors	None
Warnings	Sample concentration factor out of range

Four-Plus parameters

Alpha	0.072	08/09/2020 06:53:17	D:\Data\Customer\20I-07022_Blank standardisation.t3r
S	1.0038	08/09/2020 06:53:17	D:\Data\Customer\20I-07022_Blank standardisation.t3r
jH	0.4	08/09/2020 06:53:17	D:\Data\Customer\20I-07022_Blank standardisation.t3r
jOH	-0.3	08/09/2020 06:53:17	D:\Data\Customer\20I-07022_Blank standardisation.t3r

Titrants

0.50 M HCl	0.979835	08/09/2020 06:53:17	D:\Data\Customer\20I-07023_Blank standardisation.t3r
0.50 M KOH	1.004720	08/09/2020 06:53:17	D:\Data\Customer\20I-07020_KHP_Base standardisation using KHP.t3r

Sample

KI-ref-B concentration factor	0.096
Acid pKa 1	10.30
logP (neutral XH)	4.22
logP (X -)	3.81

Sample graphs

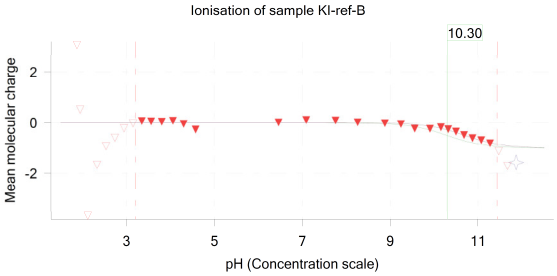

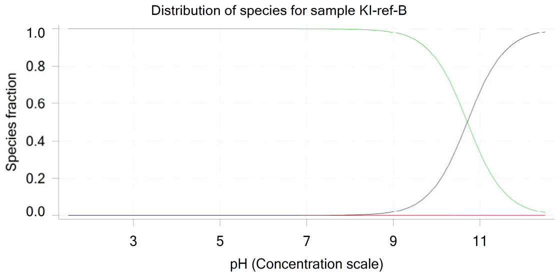

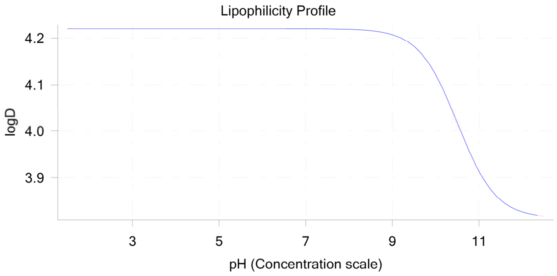

pH-metric

Sample name: **KI-ref-B**
Assay name: **pH-metric medium logP**
Assay ID: **20I-08011**
Filename: **D:\Data\Customer\20I-08011_KI-ref-B_pH-metric medium logP.t3r**

Experiment start time: **08/09/2020 06:53:17**
Analyst:
Instrument ID: **T313101**

Sample logD and percent species

pH	KI-ref-B logD	KI-ref-B KI-ref-BH	KI-ref-B KI-ref-B	KI-ref-B KI-ref-BH*	KI-ref-B KI-ref-B*	Comment
1.000	4.22	0.02 %	0.00 %	99.98 %	0.00 %	
1.200	4.22	0.02 %	0.00 %	99.98 %	0.00 %	Stomach pH
2.000	4.22	0.02 %	0.00 %	99.98 %	0.00 %	
3.000	4.22	0.02 %	0.00 %	99.98 %	0.00 %	
4.000	4.22	0.02 %	0.00 %	99.98 %	0.00 %	
5.000	4.22	0.02 %	0.00 %	99.98 %	0.00 %	
6.000	4.22	0.02 %	0.00 %	99.98 %	0.00 %	
6.500	4.22	0.02 %	0.00 %	99.97 %	0.01 %	
7.000	4.22	0.02 %	0.00 %	99.96 %	0.02 %	
7.400	4.22	0.02 %	0.00 %	99.93 %	0.05 %	Blood pH
8.000	4.22	0.02 %	0.00 %	99.78 %	0.20 %	
9.000	4.21	0.02 %	0.00 %	98.05 %	1.93 %	
10.000	4.12	0.02 %	0.01 %	83.54 %	16.43 %	
11.000	3.91	0.01 %	0.04 %	33.69 %	66.27 %	
12.000	3.83	0.00 %	0.05 %	4.83 %	95.11 %	

Carbonate and acidity

- Carbonate 0.227 mM
- Acidity error 0.763 mM

Other graphs

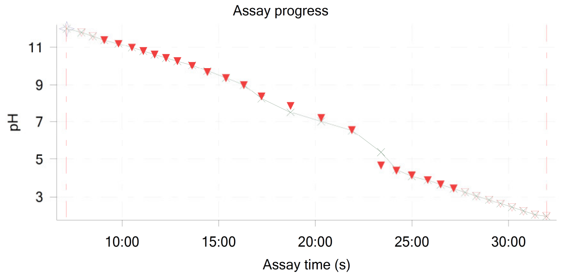

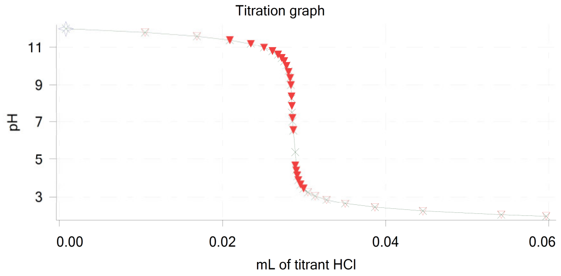

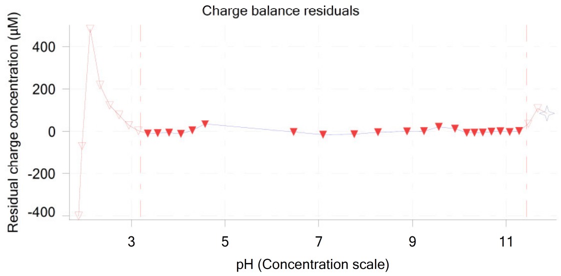

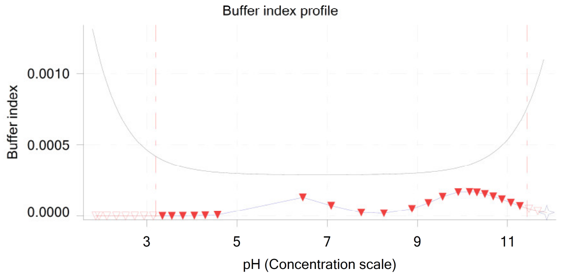

pH-metric

Sample name:	**KI-ref-B**	Experiment start time:	**08/09/2020 06:53:17**
Assay name:	**pH-metric medium logP**	Analyst:	
Assay ID:	**20I-08011**	Instrument ID:	**T313101**
Filename:	**D:\Data\Customer\20I-08011_KI-ref-B_pH-metric medium logP.t3r**		

Other graphs (continued)

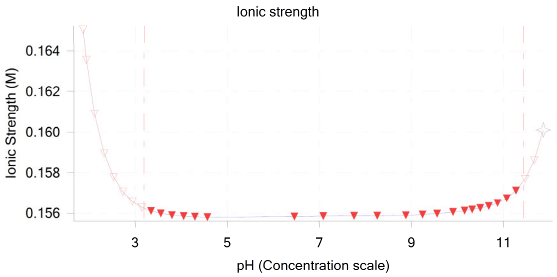

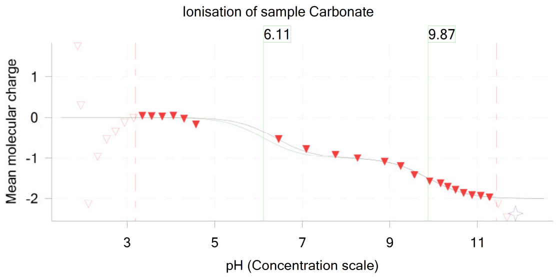

Report by: krict 08/09/2020 09:45:32 Page 3 of 3

Figure S2. pH-metric Log P of Broflanilide.

Figure S3. Ion Channels assay of 4d (KI-03066) and Broflanilide

eurofins | DISCOVERY

Release Date: 24 Aug 2020

REPORT

Study Period | 31 Jul 2020 – 21 Aug 2020

STUDY OBJECTIVE:

Client Name: KRICT
Compound Names: KI-03066, KI-Ref.B
Tested in Ion Channel GABAA α1/β3/γ2 & GlyRA1 Antagonist IonFlux Assays

STUDY INFORMATION:

- **Study code#: US034-0009484**

Eurofins Discovery
6 Research Park Drive
St Charles MO 63304
USA
T | +1 844 522 7787
F | +1 636 362 7131
www.eurofins.com

eurofins DISCOVERY

**SPONSOR INFORMATION**	Name: Sung Joon Park, Ph.D.<br>Company: KRICT<br>Address: Gajeong-ro 141, Yuseong-gu<br>Daejeon, 34114<br>Korea<br>Phone Number: +82 42-860-7085<br>Email: sjunpark@krict.re.kr

**INVESTIGATOR**	Jennifer Wesley<br>Eurofins Panlabs, Inc.<br>Tel: (636) 362-7147<br>JenniferWesley@eurofins.com

**TECHNICAL SUPPORT**	Bryan Koci, M.S.<br>Study Director, Eurofins Panlabs<br>Tel: (636) 362-7006<br>BryanKoci@eurofins.com

**STUDY APPROVAL**	*I certify that this report accurately reflects all relevant data collected in this study.*<br>Jennifer Wesley<br>*Jennifer Wesley, Associate Scientist*	*I certify that the results presented in this report were generated using materials and methods mentioned and that these results accurately reflect the raw data.*<br>Diane Werth<br>*Diane Werth, Senior Operations Manager*<br>*Quality statement*

eurofins | DISCOVERY

TABLE OF CONTENTS

TABLE OF CONTENTS 3
COMPOUND INFORMATION 4
  Test compounds 4
  Reference compounds 4
SUMMARY 5
  Ion Channels tested 5
  Study objective 5
  Measurements 5
  Significant results 5
EXPERIMENTAL RESULTS 6
  Client Compound Data Tables 6
  Estimated IC_{50} Compound Summary Table 7
REFERENCE COMPOUND RESULTS 7
  Reference Compound Table 7
DATA CALCULATION AND ANALYSIS 8
MATERIALS AND METHODS 9
STORAGE AND RETENTION OF RECORDS 10

eurofins | DISCOVERY

COMPOUND INFORMATION

Test compounds

Eurofins Compound I.D.	Client Compound I.D.	Client Reference I.D.	MW	MW + Salt	Weight received (mg)	Received condition
US034-0009484-1	KI-03066			488.45	3.1	Dry Powder
US034-0009484-2	KI-Ref.B			663.29	3.1	Dry Powder

Reference compounds

In each experiment and if applicable, the respective reference compounds were tested concurrently with the test compounds, and the data were compared with historical values determined at Eurofins. The experiment was accepted in accordance with Eurofins Standard Operating Procedure on assay validation.

Compound	Target	Mode	Concentration(s) (μM)
GABA (EC_{80})	GABAA α1/β3/γ2	Antagonist	8
Glycine (EC_{80})	GlyRA1	Antagonist	30
Bicuculline	GABAA α1/β3/γ2	Antagonist	0.032, 0.16, 0.8, 4, 20, 100
Strychnine	GlyRA1	Antagonist	0.00001<br>0.0001, 0.001, 0.01, 0.1, 1

eurofins | DISCOVERY

SUMMARY

Ion Channels tested

Ligand-Gated Ion Channels: GABAA α1/β3/γ2, GlyRA1

Study objective

Electrophysiological assays conducted to profile two (2) compounds for activities on the ion channel targets specified above using the IonFlux HT electrophysiological platform.

Measurements

Methods employed in this study have been developed and validated with reliability and reproducibility. Assays were performed under conditions described in the accompanying "Materials and Methods" section of this report

Where presented, IC_{50} values were determined by a non-linear, least squares regression analysis. Reference standards were run as an integral part of each assay to ensure the validity of the results obtained.

Significant results

Results showing an inhibition greater than 25% are considered to represent significant effects of test compounds and listed in the following tables with individual calculation results and calculable IC_{50}.

eurofins DISCOVERY

EXPERIMENTAL RESULTS

Client Compound Data Tables

GABAA α1β3γ2

Compound ID	Client Compound ID	Concentration (μM)	% inhibition		
			n1	n2	mean
US034-0009484-1	KI-03066	0.3	6.97	5.10	6.03
US034-0009484-1	KI-03066	1	7.68	8.56	8.12
US034-0009484-1	KI-03066	3	11.24	11.15	11.19
US034-0009484-1	KI-03066	10	11.91	11.25	11.58
US034-0009484-1	KI-03066	30	17.92	21.88	19.90
US034-0009484-2	KI-Ref.B	0.3	8.47	7.29	7.88
US034-0009484-2	KI-Ref.B	1	13.71	8.91	11.31
US034-0009484-2	KI-Ref.B	3	14.83	14.80	14.82
US034-0009484-2	KI-Ref.B	10	7.26	6.69	6.98
US034-0009484-2	KI-Ref.B	30	7.53	9.75	8.64
Time-Matched Vehicle Control	GABA EC_{80}	8	10.18	6.23	8.20
Time-Matched Vehicle Control	GABA EC_{80}	8	8.99	11.66	10.33
Time-Matched Vehicle Control	GABA EC_{80}	8	10.63	14.04	12.34
Positive Reference Control	Bicuculline	0.032	10.10	5.90	8.00
Positive Reference Control	Bicuculline	0.16	23.88	22.01	22.94
Positive Reference Control	Bicuculline	0.8	36.81	36.53	36.67
Positive Reference Control	Bicuculline	4	82.09	82.02	82.06
Positive Reference Control	Bicuculline	20	96.98	97.42	97.20
Positive Reference Control	Bicuculline	100	98.90	99.03	98.97

GlyRA1

Compound ID	Client Compound ID	Concentration (μM)	% inhibition		
			n1	n2	mean
US034-0009484-1	KI-03066	0.3	-1.49	-10.64	-6.07
US034-0009484-1	KI-03066	1	-1.66	-9.41	-5.54
US034-0009484-1	KI-03066	3	-8.75	-2.91	-5.83
US034-0009484-1	KI-03066	10	0.39	1.50	0.95
US034-0009484-1	KI-03066	30	4.13	8.74	6.43
US034-0009484-2	KI-Ref.B	0.3	-8.14	-2.00	-5.07
US034-0009484-2	KI-Ref.B	1	-13.15	-3.04	-8.10
US034-0009484-2	KI-Ref.B	3	-0.52	7.84	3.66
US034-0009484-2	KI-Ref.B	10	6.33	1.52	3.93
US034-0009484-2	KI-Ref.B	30	-2.53	-1.99	-2.26
Time-Matched Vehicle Control	Glycine EC_{80}	30	-2.38	2.52	0.07
Time-Matched Vehicle Control	Glycine EC_{80}	30	2.58	-2.29	0.15
Time-Matched Vehicle Control	Glycine EC_{80}	30	3.98	-0.97	1.51
Positive Reference Control	Strychnine	0.00001	-0.97	4.95	1.99
Positive Reference Control	Strychnine	0.0001	25.64	15.98	20.81
Positive Reference Control	Strychnine	0.001	50.75	53.02	51.89
Positive Reference Control	Strychnine	0.01	99.06	99.29	99.17
Positive Reference Control	Strychnine	0.1	98.87	99.02	98.95
Positive Reference Control	Strychnine	1	99.91	99.82	99.87

eurofins DISCOVERY

Estimated IC_{50} Compound Summary Table

Compound	Target	Mode	Estimated IC_{50} (μM)
KI-03066	GABAA α1/β3/γ2	Antagonist	>30
KI-Ref.B	GABAA α1/β3/γ2	Antagonist	>30
KI-03066	GlyRA1	Antagonist	>30
KI-Ref.B	GlyRA1	Antagonist	>30

REFERENCE COMPOUND RESULTS

Reference Compound Table

ITEM	Assay Name	Mode	Reference Compound	Estimated IC_{50} (μM)
CYL8053IF2	GABAA (alpha1/beta3/gamma2) Human Ion Channel Cell Based Antagonist IonFlux Assay	Antagonist	Bicuculline	1.0
CYL8056IF2	GlyRA1 Human Glycine Ion Channel Cell Based Antagonist IonFlux Assay	Antagonist	Strychnine	0.00071

DATA CALCULATION AND ANALYSIS

CYL8053IF2 GABAA IonFlux HT Antagonist Assay

Peak inward currents in response to the GABA additions in the presence of a single concentration of compound were measured. All compound data have been normalized to the baseline peak current induced by addition of EC_{80} GABA for 2 seconds:

$$Normalized\ \ Peak\ \ Current\ \ = \left(I^{Compound\,+\,GABA} / I\,GABA \right)$$

Where *I (Compound + GABA)* is the peak current induced by addition of test compound + EC_{80} GABA after 30 seconds incubation of test compound, *I GABA* is the baseline peak current induced by addition of EC_{80} GABA.

All data were first exported to an Excel compatible data file and then analyzed using Graph Pad Prism software.

CYL8056IF2 GlyRA1 IonFlux HT Antagonist Assay

Peak inward currents in response to the Glycine additions in the presence of a single concentration of compound were measured. All compound data have been normalized to the baseline peak current induced by addition of EC_{80} Glycine for 2 seconds:

$$Normalized\ \ Peak\ \ Current\ \ = \left(I^{Compound\,+\,Glycine} / I\,Glycine \right)$$

Where *I (Compound + Glycine)* is the peak current induced by addition of test compound + EC_{80} Glycine after 30 seconds incubation of test compound, *I Glycine* is the baseline peak current induced by addition of EC_{80} Glycine.

All data were first exported to an Excel compatible data file and then analyzed using Graph Pad Prism software.

eurofins | DISCOVERY

MATERIALS AND METHODS

IonFlux Protocols

CYL8053IF2 GABAA IonFlux HT Antagonist Assay

All recordings were obtained from a holding potential of -60 mV.

The compound addition sequence that was used for all additions was the same for all assays. One addition of the EC_{80} concentration of GABA was added to establish baseline response. Each test concentration of compound was applied for 30 seconds followed by the addition of EC_{80} GABA in the presence of the compound for 2 seconds. The process was repeated with the next ascending concentration of test compound, up to three (3) concentrations per experimental pattern.

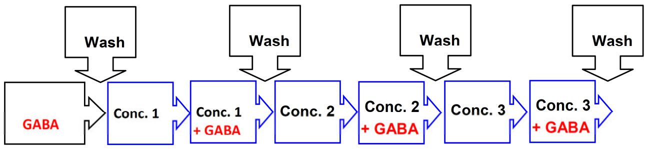

Key

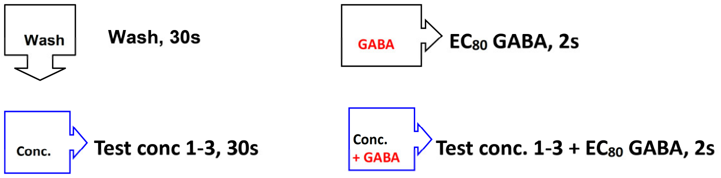

eurofins DISCOVERY

CYL8056IF2 GlyRA1 IonFlux HT Antagonist Assay

All recordings were obtained from a holding potential of -60 mV.

The compound addition sequence that was used for all additions was the same for all assays. One addition of the EC_{80} concentration of Glycine was added to establish baseline response. Each test concentration of compound was applied for 30 seconds followed by the addition of EC_{80} Glycine in the presence of the compound for 2 seconds. The process was repeated with the next ascending concentration of test compound, up to three (3) concentrations per experimental pattern.

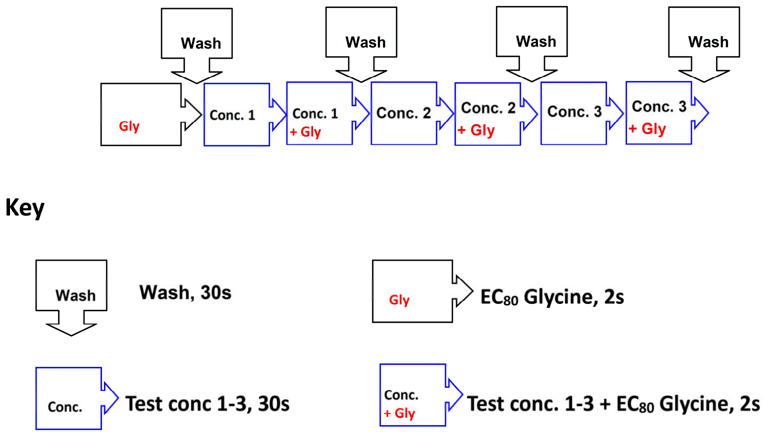

STORAGE AND RETENTION OF RECORDS

Documents generated during the performance of the study will be archived by Eurofins Discovery for a period of time after study completion (this period of time is dependent on each individual site policy). The access to the archives is restricted to authorized employees only.

© 2020 by the authors. Licensee MDPI, Basel, Switzerland. This article is an open access article distributed under the terms and conditions of the Creative Commons Attribution (CC BY) license (http://creativecommons.org/licenses/by/4.0/).
